# Self-, other-, and joint monitoring using forward models

**DOI:** 10.3389/fnhum.2014.00132

**Published:** 2014-03-25

**Authors:** Martin J. Pickering, Simon Garrod

**Affiliations:** ^1^Department of Psychology, University of EdinburghEdinburgh, UK; ^2^Institute of Neuroscience and Psychology, University of GlasgowGlasgow, UK

**Keywords:** monitoring, production, comprehension, dialogue, forward models

## Abstract

In the psychology of language, most accounts of self-monitoring assume that it is based on comprehension. Here we outline and develop the alternative account proposed by Pickering and Garrod (2013), in which speakers construct forward models of their upcoming utterances and compare them with the utterance as they produce them. We propose that speakers compute inverse models derived from the discrepancy (error) between the utterance and the predicted utterance and use that to modify their production command or (occasionally) begin anew. We then propose that comprehenders monitor other people’s speech by simulating their utterances using covert imitation and forward models, and then comparing those forward models with what they hear. They use the discrepancy to compute inverse models and modify their representation of the speaker’s production command, or realize that their representation is incorrect and may develop a new production command. We then discuss monitoring in dialogue, paying attention to sequential contributions, concurrent feedback, and the relationship between monitoring and alignment.

## Introduction

Most psycholinguists discuss monitoring as a component of a theory of speaking (or language production), and therefore refer to it as *self-monitoring*. In fact, people also perform *other-monitoring*, when they monitor their partners during comprehension. In this paper, we propose related accounts of self- and other-monitoring and combine them in an account of monitoring in dialogue. Our proposals apply Pickering and Garrod (2013) integrated account of language production and comprehension, and assume that people predict both their own and their partners’ utterances.

In language production, the dominant view of self-monitoring involves comprehension (Levelt, 1983, 1989). Speakers monitor their overt speech and sometimes correct errors or infelicities after they occur, or they monitor an internal representation of their planned speech (usually couched in terms of phonology or phonetics) and sometimes correct errors or infelicities in that representation. Alternative accounts assume that speakers monitor by detecting conflicts or degraded representations during the production process (e.g., Nozari et al., 2011). In contrast, we argue that self-monitoring critically involves prediction within the production system and a process of comparing predictions with “implemented” representations constructed during production.

In addition, people can clearly monitor other people’s speech as well as their own. We propose that comprehenders make predictions about speakers’ utterances and compare these predictions with the speakers’ actual utterances. We refer to this process as *other-monitoring*. Comprehenders can then query or correct speakers’ utterances depending on the circumstances. Our account of other-monitoring makes use of Pickering and Garrod (2013) argument that comprehenders’ predictions are typically based on their own production system (using what we term “prediction-by-simulation”).

Up to this point, our focus is on monologue or narratives, where production and comprehension are fairly distinct. The final part of the paper concerns interactive dialogue, in which contributions are tightly interwoven, and hence self- and other-monitoring are also interwoven. Other-monitoring, in particular, now plays a quite different and more crucial role than it does in monologue or narrative comprehension.

## Self-monitoring

As we have noted, self-monitoring is traditionally explained using the comprehension system. Levelt (1989) account assumes that speakers monitor via an “outer loop”, whereby they hear what they say, and the process of comprehension is essentially the same as comprehending another person’s speech. But speakers also monitor via an “inner loop”. Levelt (1983) considers a speaker who utters *to the ye- to the orange node* when attempting to describe an orange node as part of a route around a colored network. The comprehension-based account proposes that the speaker constructed a representation of *yellow* at a phonological (or phonetic) level, comprehended it (using the comprehension system), realized that the resulting meaning (i.e., the color yellow) did not match the situation or intended meaning, and reformulated. In other words, the speaker monitored an “inner” representation. One classic piece of evidence comes from Motley et al. (1982), who primed participants with word pairs beginning with particular consonants (e.g., *k–t*). Participants then read a word pair in which the consonants were reversed (*tool-kits*) and they sometimes produced a taboo error (*cool-tits*). Participants who did not produce a taboo word generated a heightened galvanic skin response, associated with emotional arousal.

Importantly, Hartsuiker and Kolk (2001) developed a computational implementation of Levelt (1989) account that successfully simulated the distribution of error-to-cutoff and cutoff-to-repair intervals in relation to speech rate. However, they assumed that speakers construct the input for “inner loop” monitoring 250 ms before articulation and therefore provide enough time for the comprehension system to detect anomalies and interrupt speech. But more recently, Indefrey and Levelt (2004) estimated that phonetic encoding and articulation take about 145 ms, and hence comprehension-based monitoring would have no more than 145 ms available (and if some of the relevant representations are phonetic, it might have even less time). In this time, the speaker would have to perform most of the processes involved in word comprehension (which takes at least 145 ms; e.g., Sereno et al., 2003), compare the meaning to the intended utterance, and reformulate. However, it is possible that speakers might “buffer” linguistic material by delaying phonetic encoding and articulation on utterances involving pre-articulatory repairs (perhaps as a result of constructing semantic, syntactic, and phonological representations more quickly than they can articulate them). One problem for this proposal is that speeding up articulation ought to interfere with monitoring and repair, but in fact people repair more quickly, when they speak faster (see Postma, 2000).

Moreover, speakers would face the extreme complexity of comprehending an earlier part of a sentence with the external loop and a later part with the internal loop (Vigliocco and Hartsuiker, 2002; Nozari et al., 2011). Finally, many patients show a dissociation between comprehension and self-monitoring (Nozari et al., 2011). Presumably there must be some form of comprehension-based monitoring of external speech (though it may be partly inhibited by prediction and reafference cancellation); the evidence for “inner-loop” comprehension-based monitoring is much less clear.

Alternative accounts do not use the comprehension system to monitor (e.g., Laver, 1980; Schlenk et al., 1987). For example, Laver assumed that speakers may monitor by detecting problems with the production process itself. In Levelt (1983) example, the speaker might construct representations of *yellow* that are sufficiently accurate to trigger production processes but which have significant discrepancies from “canonical” representations. On this basis, the speaker determines that they may not be accurate and interrupts production. Although this account has not been extensively addressed, it is related to MacKay (1987) Node Structure Theory, which assumes that such representations appear erroneous because they have not been previously constructed (i.e., are new to the system). More recently, Nozari et al. (2011) suggested that speakers detect conflicts between alternative representations (see Botvinick et al., 2001). Levelt’s (1983) speaker might have constructed a representation for *yellow* but also a (weaker) representation for *orange* and realized that these representations conflicted.

A rather different approach assumes that speakers construct predictions of what they are about to say before they speak and then compare those predictions with their actual implementation of speech. Following the action-control tradition, such predictions make use of *forward models* (see Wolpert, 1997). For example, if I decide to move my hand to a particular location, I combine my intention, my hand’s position in relation to the environment, and my experience of the outcome of previous similar intentions to construct a representation of my predicted hand movement. I might predict that my hand will end up 500 mm from my body and 30° left of my midline, in 300 ms time. Importantly the prediction will be ready in considerably less than 300 ms, before the movement. The prediction can then be compared with the actual movement “when it comes in”.

Such predictions can be quite accurate because I have so much experience of moving my hand. However, people’s actions are not entirely accurate; in this case my hand might end up 31° left of my midline. If so, I use the discrepancy (1° to the left) as input to an *inverse model* that is fed back to modify my intention. If I then attempt to perform the same act again, I am likely to construct a more accurate forward model and also to perform a more accurate act. It is through computing such forward and inverse models that I first learnt to control the movement efficiently (see Wolpert et al., 2001). Note that we are simplifying by assuming that the forward model only plays a role at the point at which the action is completed. But in fact this is not the case—the forward model is available on-line, at all points during the movement. Importantly, in combination with the inverse model, it is used to “shape” the movement, thus reducing the discrepancy between actual and predicted movement. Finally, Wolpert et al. assume the existence of multiple pairs of forward and inverse models, with the agent continuously attempting to determine which pair is most accurate. The “error” is of course the discrepancy between the best (selected) model and the behavior (i.e., the error is minimized).

Some researchers have proposed related accounts to explain phonological and articulatory aspects of speech production. Thus, Tourville and Guenther (2011) described their DIVA (Direction of Velocities of Articulators) model (and its extension, the Gradient Order DIVA model), which uses auditory and somatosensory forward modeling as part of a computationally explicit and neurobiologically grounded account of speech production and acquisition. Similarly, Hickok et al. (2011) proposed a state feedback control account in which an internal model generates predictions about the state of the vocal tract and predictions about the sensory consequences of articulation. Both of these proposals are focused on prediction (and adaptation) at “low” levels and do not attempt to generalize to syntactic and semantic aspects of self-monitoring.

In contrast, Pickering and Garrod (2013) proposed that people predict their own utterances at the full range of linguistic levels and use those predictions in self-monitoring. Before speaking, people construct an intention (called the production command) and then use forward models to predict characteristics of their utterance based on this intention and their memory of the outcome of similar intentions in the past. The account is similar to Wolpert et al. (2001), except that we assumed that people make predictions at different levels of representation, concerned with meaning, grammar, or sound. Thus, the speaker might predict that she will produce a noun, a word referring to something edible, and/or a word beginning with a vowel.

The speaker can then match her predicted percept of the utterance against her actual percept, using self-monitoring, and use the discrepancy as input to an inverse model that modifies the production command and hence the unfolding utterance. The comparison is direct because the predicted percept is in the same format as the percept, and therefore no analysis of the percept is necessary before comparison. This contrasts with comprehension-based monitoring, in which the utterance has to be analyzed before comparison is possible. Nozari et al.’s (2011) conflict-detection account also allows rapid determination of potential production errors, but does not in itself provide any basis for correction (Note, however, that Botvinick et al., 2001, used conflict-detection to bias actions toward correct choices; it remains to be seen whether an equivalent account can be developed for language production). According to our account, the speaker of *to the ye- to the orange node* predicted some aspects of the representations associated with *orange* before uttering *ye-*, for example that it begins with the phoneme ɒ. She then produced the representations associated with the phoneme *j*, realized that they did not match, and reformulated. More specifically, Pickering and Garrod (2013) proposed that speakers generate a production command *i*(*t*) that initiates two processes. First, it provides the input for the production implementer, which contains the mechanisms involved in production itself, and outputs an utterance $p\underline{\left[sem,syn,phon](t\right)}$, a sequence of sounds that encodes semantics, syntax, and phonology. The speaker then uses the comprehension implementer to construct the utterance percept $c\underline{\left[sem,syn,phon](t\right)}$, the perception of the sequence of sounds that encodes semantics, syntax, and phonology. Second, an efference copy of *i*(*t*) feeds into a forward production model, a computational device that outputs the predicted utterance $\hat{p}\underline{{\left[sem,syn,phon\right]}_{B}(t)}$. This in turn feeds into the forward comprehension model, which outputs the predicted utterance percept $\hat{c}\underline{{\left[sem,syn,phon\right]}_{B}(t)}$. The utterance percept and predicted utterance percept can be compared by self-monitoring. Pickering and Garrod (2013) stopped at this point, and did not discuss the process of comparison, its effects, the mechanisms involved in reformulation, or the information that feeds into the production command itself.

The process of comparison involves determining the discrepancy (error) between the utterance percept $c\underline{\left[sem,syn,phon](t\right)}$ and the predicted utterance percept $\hat{c}\underline{\left[sem,syn,phon](t\right)}$, which we call $\underline{\Delta [sem,syn,phon](t)}$ (i.e., $\underline{\Delta [sem,syn,phon](t)}=\underline{c[sem,syn,phon](t)}-\underline{\hat{c}[sem,syn,phon](t))}$. In practice, this is “unpacked”, so that the speaker computes separate linguistic levels, for example the phonological percept $c\underline{\left[phon](t\right)}$ the predicted phonological percept $\hat{c}\underline{\left[phon](t\right)}$ and their discrepancy $\underline{\Delta [phon](t)}$. We assume that the utterance percepts and predicted utterance percepts can be directly compared and therefore the discrepancy can be computed. So in Levelt’s (1983) example, the phoneme is *j*, the predicted phoneme is ɒ, and the discrepancy is the difference between *j* and ɒ. In this case, the phonemes are very different (e.g., sharing few phonetic features) and so the discrepancy is large; in other cases (e.g., *b* vs. *d*), the discrepancy would be smaller. Importantly, the speaker can represent this discrepancy without computing all aspects of phonetics, which might of course not be represented in the forward model.

**Figure 1:**
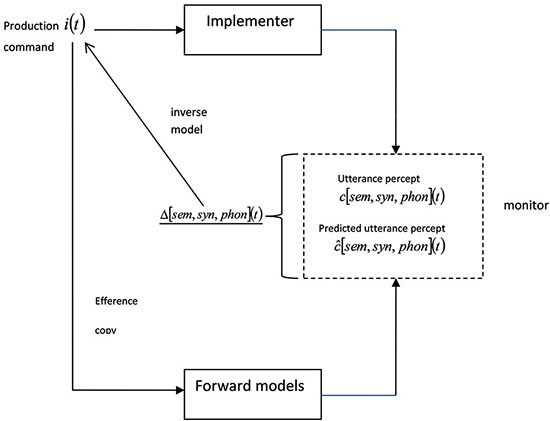
**Self-monitoring using forward models.** The implementer uses the production command to construct the utterance percept. The forward models use an efference copy of the production command to construct the predicted utterance percept. The monitor computes the discrepancy between the utterance percept and the predicted utterance percept, which can then be used (as an inverse model) to modify the production command, or assist in the construction of a new production command.

We propose that the discrepancy, $\underline{\Delta [sem,syn,phon](t)}$, has a different effect when it is high (large) vs. low (small). When it is high, the correction would go beyond the scope of the current forward-inverse model pairing, and the speaker has to construct a new production command (or give up). But when it is low, the speaker can use his inverse model to correct subsequent predictions directly. Specifically, the monitor feeds the discrepancy back to the production command and uses it to modify the command (see Figure 1). If this occurs quickly enough, the speaker simply changes his utterance on-line, perhaps pausing or lengthening the utterance. In other cases, the signal from the inverse model does not arrive until the implementer is too far advanced, and the speaker produces some or all of the originally planned utterance and then reformulates, as in Levelt’s (1983) example.

Note that the representation of the utterance percept in Figure 1 is a simplification (as discussed in Pickering and Garrod, 2013). The semantic component of the utterance percept (i.e., $c\underline{\left[sem](t\right)}$) is constructed before the syntactic component, which is in turn computed before the phonological component. The predicted utterance percept is similarly divided into semantic, syntactic, and phonological components. Thus it is possible to perform pre-response monitoring, for example by comparing the semantic component with the predicted semantic component “early” in the production process, well before the utterance is produced. In addition, speakers should notice their own semantic errors early and their phonological errors late, in contrast to comprehension-based monitoring accounts (Levelt, 1983), in which speakers comprehend the phonology of their prepared utterance before determining its semantics.

Notice that this account assumes that error-detection and repair involve “revisiting” the production command (i.e., the intention underlying speaking) and do not simply involve “patching up” a stage in production (e.g., phonology). This is analogous to the correction of motor movement—when an agent creates an action plan to move her arm to *X* and in fact moves it to *Y*, the discrepancy (*Y–X*) is used to modify the action plan. In other words, monitoring and repair involve intentional mechanisms. It is, however, possible that an inverse model generated from comparing, say, the phonological percept and the predicted phonological percept might feed back to a stage of implementation, for example concerned with semantics. However, we propose that such an inverse model would be accompanied by an inverse model feeding back to the production command (as in Figure 1).

There is considerable evidence for the use of forward models in language production (see Pickering and Garrod, 2013). In an magnetoencephalography (MEG) study, Tian and Poeppel (2010) had participants imagine producing a syllable, and found the same rapid response in auditory cortex as when they articulated (see also Tian and Poeppel, 2013). This suggests that even when imagining speaking, people use a forward model to construct the predicted utterance percept incorporating phonological information. In another MEG study, Heinks-Maldonado et al. (2006) found that the M100 (i.e., occurring about 100 ms post-stimulus) was reduced when people spoke and concurrently listened to their own unaltered speech vs. a pitch-shifted distortion of the speech.

Tourville et al. (2008) provided evidence about the process of error correction using altered speech. They had participants read monosyllabic words aloud and distorted feedback by shifting the first formant up or down on a small proportion of trials. They found that participants shifted their speech in the opposite direction very rapidly, within about 100 ms for downward shifts. Pickering and Garrod (2013) noted that such rapid compensation provides strong evidence that self-monitoring involves prediction within the production system. Speakers construct a predicted utterance percept based on the efference copy of their production command, which we assume to be accurate—say, $\hat{c}\underline{\left[F1](t\right)}=x Hz$ (We use *F*1 to refer to first formant, and regard it simplistically as an aspect of *phon*). On the trials in which the first formant was decreased by 30%, c[F1](t)¯=0.7x Hz, and hence Δ[F1](t)¯=−0.3x Hz. This discrepancy is fed into the production command using the inverse model (see Figure 1), and the production command can then be modified on-line so that F1 is shifted upwards (Presumably the feedback is targeted at the aspect of the production command that deals with F1, so there is no need to revisit other aspects of the production command). We can similarly explain effects of adaptation to ambient noise (Lombard effects; e.g., Lane and Tranel, 1971).

In language production, most monitoring leads to low (“small”) discrepancies, because speakers tend to prepare utterances that are largely compatible with their intention (production command). This means that the discrepancy can be used to modify the production command (In Levelt’s example it is presumably possible to correct the semantics and then recompute phonology). As an example, Boland et al. (2005) had participants plan descriptions of objects (e.g., *dark blue square*) which they then modified before speaking. On the basis of their results, they argued that speakers could delete words (e.g., producing *blue square*) from their current plan, thus revising their plan rather than starting afresh.

However, it is also possible that the discrepancy is “high”, and the predicted utterance percept is sufficiently unrelated to the utterance percept that the speaker is forced to construct a new production command. If so, the speaker has to appeal to “general knowledge” (thinking) and leaves the language processing mechanism. As an example, consider the effects of shifting F1 considerably more than 30% using Tourville et al.’s (2008) paradigm. At some point, participants would presumably stop compensating for the discrepancy. They might reason about the cause of the discrepancy and make an explicit decision about how to react. Such a process would not be internal to language processing.

A related situation occurs when a relevant aspect of the environment changes as the speaker is referring to it. For example, a football commentator might be describing a particular player’s possession of the ball when he is suddenly tackled. In one set of experiments, participants named pictures which occasionally changed into different pictures during naming (Hartsuiker et al., 2005; Tydgat et al., 2012; cf. Van Wijk and Kempen, 1987). Participants sometimes interrupted themselves while naming the first picture. In at least these cases, they replaced their production command on the basis of a change in the world (i.e., external to Figure 1). Again, the discrepancy is large (though note that the response may not be regarded as an error).

Note that there is much evidence that the implementer constructs parallel representations which in some cases feed into later stages of processing (e.g., Peterson and Savoy, 1998). Moreover, activation appears to cascade across stages, so that speakers begin to construct representations at later levels before representations at earlier levels are complete (e.g., Goldstein et al., 2007; McMillan and Corley, 2010). At least some of the time, the implementer therefore appears to entertain more than one alternative representation at the same level or at different levels. Such cascading activation is not problematic for forward models. In fact, Pickering and Garrod (2013) assumed that speakers simultaneously construct forward models corresponding to different levels of representation. At a particular level, they could construct a single forward model corresponding to one implementation. Alternatively, they could entertain multiple forward models in parallel at a particular level, as assumed in at least one account of motor control (MOdular Selection And Identification for Control (MOSAIC; Haruno et al., 2001)). If so, the speaker could use the smallest prediction error as input to the inverse model. For example, if I predict both *couch* and* sofa*, and I implement *conch*, the input to the inverse model would be the (phonological) discrepancy between *conch* and *couch* (Parallel prediction is likely to be more central to comprehension, as we shall see).

Finally, we note that conflict-monitoring accounts (e.g., Nozari et al., 2011) may be consistent with forward modeling if the detected conflict is between predicted and implemented representations. A particular advantage of involving forward modeling is that the forward model is likely to be accurate (as it is the result of learning intention-outcome mappings), and can therefore guide the process of correction. In addition, it has the benefit of extending to other-monitoring with the same basic system (see below).

## Other monitoring

Just as speakers can monitor their own utterances, so comprehenders can monitor speakers’ utterances. For example, people can reformulate to remove their own speech errors or infelicities. In a similar way, they can propose corrections of other people’s utterances or query those utterances (in a way that perhaps prompts the original speaker to reformulate). Under some circumstances, such reformulations (and queries) are very common and spontaneous, as in parent-child dialogue (e.g., Chouinard and Clark, 2003); they are of course also central to instructional dialogue. We argue that comprehenders constantly perform other-monitoring, in that they predict speakers’ utterances and compare those predictions with their actual utterances as they unfold (for a comparable proposal, see Van de Meerendonk et al., 2009). They can then use any discrepancies in various ways including other-repair. On our account, other-monitoring is related to self-monitoring. Below, we outline this account, and then contrast cases where comprehenders cannot usefully respond (e.g., listening to the radio) and cases where they do (as may happen when listening to a co-present narrator).

Let us return to the example of hand movement. When I see you starting to move your hand, I use forward modeling to predict where your hand will end up, before you move your hand, and then compare this with your actual movement. Pickering and Garrod (2013) proposed that there are two “routes” to prediction. Their focus is on *prediction-by-simulation*, which is used for actions performed by other people. We have already noted that people develop forward models in order to learn and control their actions, and use these models to predict action outcomes. Such forward models are therefore available to predict other people’s actions as well. To do this, Pickering and Garrod proposed that people covertly imitate each other’s actions and derive the action command underlying their upcoming action (while compensating for differences between them). They then use this action command to construct a forward model of the predicted outcome of the action. To predict your hand movement, I covertly imitate your movements and then use the result of this imitation to construct a prediction as when predicting my own hand movement (e.g., Haruno et al., 2001).

Pickering and Garrod (2013) also proposed that people use *prediction-by-association*. This route to prediction is based on perceptual experiences alone. Thus, I predict on the basis of experience of other people (including you) moving their hands in the past. In fact, I can predict the movement of inanimate objects in the same way. Prediction-by-association does not have access to the processes involved in learning and correcting one’s own actions, though it can instead benefit from correction of perceptual predictions. We assume that people combine both types of prediction in action perception.

As language comprehension is a form of action perception, it can also involve prediction-by-simulation. The comprehender *A* hears the start of *B’*s utterance (e.g., *I want to fly my*), and covertly imitates it, then uses an inverse model and context (which constitutes information about the differences between *A* and *B’*s language mechanisms) to derive the production command that *B* would use to produce the utterance so far. *A* then runs it ahead to derive the production command for the next part of the utterance (here, *kite*). *A* then constructs an efference copy of this command to input into the forward models and output the predicted utterance percept—in this case, components of the experience associated with saying *kite*. Meanwhile, *B* continues with his utterance, either saying *kite* or some other word. *A* can then compare the predicted with the actual utterance using other-monitoring.

**Figure 2:**
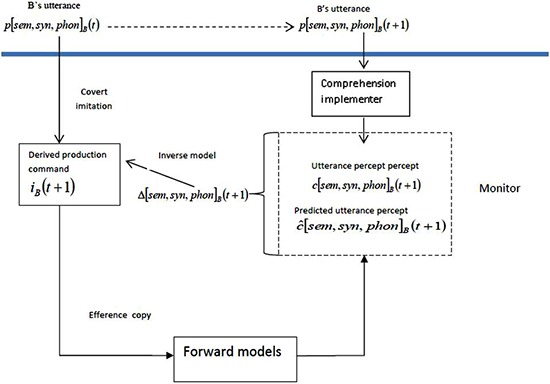
**Other-monitoring using forward models.** Information above the line (and underlined) refers to *B*, and indicates the representations underlying the utterance at time *t* and the upcoming utterance. The arrow is dotted because the relationship between the two representations is not causal for *A*. *A* uses covert imitation, including access to the inverse model and context (i.e., differences between *A* and *B*) to derive *B’*s production command, and then uses forward models to derive the predicted utterance percept. *A* then comprehends *B’*s utterance and compares the utterance percept and the predicted utterance percept. The output of the comparison is the discrepancy between the predicted and actual percepts, and it provides the input to the inverse model that can be subsequently used to modify the derived production command.

We explain this process in more detail using Figure 2, which is based on Figure 6 from Pickering and Garrod (2013), but both simplified and extended, as it considers the output of the monitor. We refer to the discrepancy between the comprehender’s predicted utterance percept $\hat{c}\underline{{\left[sem,syn,phon\right]}_{B}(t+1)}$ and the comprehender’s utterance percept $c\underline{{\left[sem,syn,phon\right]}_{B}(t+1)}$ as $\underline{\Delta {\left[sem,syn,phon\right]}_{B}(t+1)}$. This is the same as in production, except that we have added the subscript *B* to refer to the speaker.^1^

In one sense, the use of the discrepancy is the same in self- and other-monitoring. But the comprehender has much less solid information about the speaker’s production command than the speaker does. This means that the comprehender’s prediction will often be significantly inaccurate—big discrepancies are much more common than in production. Hence it will often not be possible to use the inverse model to modify the derived production command. Informally, the comprehender fails to work out what the speaker “means” and leaves the language processing system, as discussed below.

Moreover, the effect of modifying or reformulating the production command is rather different in comprehension. Self-monitoring enables speakers to check that their communicative intentions have been correctly implemented. In other words, the predicted utterance directly reflects the speaker’s intention and any discrepancy in the perceived utterance can be corrected to conform to this intention. (We assume that mature speakers of a language rarely make erroneous predictions about their own utterances.) By contrast, other-monitoring enables listeners to check that their predictions about the other’s utterance correspond (at some level) with what the other actually says. Any discrepancy can then be used to do two things. First, it can lead to the listener updating his prediction of the upcoming utterance by modifying the next derived production command (as indicated above). This rapid updating therefore helps the listener make predictions as quickly and effectively as possible. Second, the discrepancy can be used to indicate the degree to which the other’s utterance conforms to the listener’s prediction (in other words, the extent to which it makes sense for the listener). If the discrepancy is large, the listener realizes that his prediction is very different from the speaker’s utterance. This provides a cue to the listener to seek the reason for the discrepancy (“I’ve been misinterpreting her—I wonder why?”).

An additional complication is that the listener *A* may vary in his confidence about both *B’*s utterance and about his ability to predict *B’*s utterance. For example, if *B’*s utterance is partly obscured by noise, then *A* will be less confident about what he heard than otherwise. In this case, *A* will tend to place more faith in the predicted utterance percept. Similarly, if *A* is unsure about *B’*s background knowledge (e.g., whether *B* shares *A’*s cultural expectations about things that people tend to fly), then *A* may be less confident about predicting *B’*s utterance (in this case, the word *kite*) than otherwise. In this case, *A* will tend to place more faith in the actual utterance percept. To model this, we assume a variable, CON_U_, to refer to confidence in the utterance. A complete account would assume another variable, CON_P_, to refer to confidence in the prediction (but we do not discuss this further).

Under conditions with little background noise, we assume that CON_U_ is high, and so the inverse model (and hence the modification to the production command) is largely dependent on the utterance percept.^2^ Under less clear conditions when background noise is high or the utterance itself is unclear (i.e., CON_U_ is low), *A* may “assume he misheard” and not modify his production command. Lack of clarity is much more likely for a single phoneme than a whole word (or phrase), and it may be that normal speech is perceived as “noisy” from the point of view of the phoneme. In phoneme-restoration experiments (Warren, 1970), a word is strongly predicted (both because it fits the context and because people tend to utter complete words). The comprehender “sticks with” the predicted utterance percept (the phoneme) and the discrepancy is (near to) zero. Thus the missing phoneme does not cause the comprehender to modify his production command. Interestingly, it is in just such “noisy” non-ideal conditions that motor areas become most active during speech perception (Scott et al., 2009; Adank, 2012; D’Ausilio et al., 2012). This is consistent with listeners relying more on prediction-by-simulation when CON_U_ is low than when it is high.

We now focus on cases where confidence in both the prediction and the utterance are high. Considering the first consequence of other-monitoring, it will often be possible to use the discrepancy to modify the derived production command “internally”. This is the case when the discrepancy is low, a situation which is less ubiquitous than in language production but which we assume is still typical. The derived production command will then be modified to follow the utterance percept. After *I’m going to ride my*, the comprehender *A* predicts *bike*. However, the speaker *B* may actually say */s/* rather than */k/* (at time *t* + 1). At this point, *A* predicts that *B* has uttered the start of *bicycle* rather than *bike*. The comprehender *A* may then modify his derived production command using the discrepancy between these phonemes and use it to predict that *B* will now say the final phonemes of *bicycle* (at *t* + 2). Considering the second consequence of other-monitoring, the listener is unlikely to notice the small discrepancy between predicted and actual utterance (or realize that his prediction has changed)—changes internal to the derived production command do not (normally) result in awareness.

But what happens when the discrepancy is sufficiently high that the comprehender cannot modify the derived production command? We propose that he has to construct a new derived production command, and to do this, he must make use of mechanisms additional to those included in Figure 2. This means that updating of the predictions will tend to be slower than is the case for small discrepancies. In addition, comprehenders will often seek an explanation for the discrepancy (as noted above). There are two possible mechanisms for constructing a new derived production command: The comprehender can work out in his head what is going on (internal modification), or the comprehender can query the speaker (external modification). We consider these in turn.

### Internal modification

If other-monitoring produces a sufficiently large mismatch between predicted and actual utterance, the comprehender cannot directly update the derived production command and is therefore forced to draw on information from outside the language system by appealing to general knowledge. Informally, he asks “what can the speaker have meant?” When listening to the radio (for example), he has to hope that he can answer this question sufficiently quickly that he can “catch up”, or else must accept a gap in his understanding. When listening to a podcast (or while reading), he can also decide to resample by replaying some or all of what he has heard (or instigating a regressive eye-movement in reading).

Unless resampling is possible, the comprehender attempts to determine what he does not understand about the situation—in which case he appeals to general knowledge—or the knowledge about the speaker—in which case he appeals to his theory of mind. In the latter case, he “mentalizes”: he uses explicit reasoning about the speaker and his assumptions about theory of mind to determine how to interpret the utterance. Even if the comprehender can in principle resolve the discrepancy internally, a large discrepancy may take a lot of time to resolve and general knowledge or theory of mind may therefore be brought to bear (without it being strictly necessary).

To a large extent, other-monitoring is concerned with determining whether the comprehender’s estimate of the speaker’s communicative intention (i.e., derived production command) is correct. It therefore engages mechanisms associated with theory of mind, and hence a large discrepancy is likely the result of a disconnect between a belief about the speaker’s intention and a realization of that intention. In such cases, we expect extensive activation of networks associated with theory of mind (in order to help resolve the discrepancy).

In relation to this, Bašnáková et al. (2013) carried out an fMRI study contrasting direct and indirect replies to questions. People listened to utterances such as *It is hard to give a good presentation* as either a direct reply to a question (e.g., *How hard is it to give a good presentation?*) or an indirect reply (e.g., to *Did you like my presentation?*). Listening to indirect in contrast to direct replies activated a large frontal and medial prefrontal network, including brain regions previously implicated in mentalizing and empathy (medial frontal cortex (MFC), right temporo-parietal junction (TPJ), and the anterior insula) (see Amodio and Frith, 2006). Understanding an indirect reply requires determining the pragmatic relationship between it and the question, and pragmatics involves the beliefs, knowledge and intentions of the participants in the speech event (Levinson, 1983). So when a comprehender interprets a reply directly, but then realizes that it does not make sense, he is forced to engage theory of mind in reanalysis. The same would be true if the comprehender changes from one indirect interpretation of an utterance to another.

We propose that comprehenders quite often fail to predict that they will get a particular form of indirect response to a question. When this happens, the discrepancy between the predicted and actual utterance requires the comprehender to involve mechanisms outside the language system. In particular, he revises the production command and makes use of mentalizing networks to do so.

Some evidence suggests that difficulty in language comprehension leads to activation of brain structures associated with conflict resolution that reflects the need to select among competing alternatives (see Novick et al., 2010). Such difficulty can be due to conflict between the utterance percept and top-down processes which presumably help generate the predicted utterance percept (see also Slevc and Novick, 2013). January et al. (2009) found that comprehension of garden-path sentences (in which the syntactic structure turns out to be incompatible with the predicted structure) led to similar activation of the Left Inferior Frontal Gyrus (LIFG) to that found in a Stroop task (which involves general-purpose conflict). We propose that comprehenders are unable to modify the production command quickly to deal with the new syntactic structure and hence call on non-linguistic mechanisms that are used in conflict resolution. In conclusion, comprehenders may need to revise the production command because of a discrepancy in pragmatic interpretation or syntactic analysis (and perhaps for other reasons as well).

### External modification

In many cases, addressees can respond to the speaker. This is perhaps analogous to a perceiver moving his eyes or head to interpret an ambiguous percept. Of course, speakers are at least potentially affected by addressees’ responses (e.g., Krauss and Weinheimer, 1966; Bavelas et al., 2000), so such responses may prove effective in improving the speaker’s contribution. Types of response include looks of puzzlement or surprise, queries, acknowledgments, and contributions to the narrative (see Clark, 1996).

An illustrative example comes from Drew (1997):^3^

(1) Hal: an’ Leslie ’t was marv’lous (.) D’you know he had (.) **forty nine g’rillas**. .hh th-there. (b) (.) br[eeding in ( )

Lesley: [pf- f- **Forty nine wha:t?**

Hal: **G’rillas.**

Lesley: hh Oh ye-s?

While listening to Hal’s first utterance, Lesley presumably does not predict that Hal will refer to gorillas at that point. Instead, she predicts that Hal will refer to something that people typically have forty nine of (e.g., small or cheap things). She then hears *g’rillas* and presumably interprets the word correctly (via the implementer) and has high confidence in this interpretation (i.e., CON_U_ is high). However, the discrepancy between her semantic prediction and the semantics of gorillas is too great to resolve by modifying the derived production command. She could have attempted to construct a new derived command internally but this might not have been successful. She instead queried Hal by uttering *Forty nine wha:t?* (It may be that Lesley heard enough of *breeding* to determine that Hal in fact intended to refer to gorillas and therefore decided to check Hal’s utterance.) This external modification specifically identifies the locus of difficulty and thus allowed Hal to utter *g’rillas* again. Before Hal says this, Lesley is likely to predict that Hal will refer to gorillas and so there is very little discrepancy between Lesley’s predicted utterance percept and the actual percept. At this point, Lesley appears to indicate understanding (*Oh ye-s?*).

In conclusion, Lesley was unable (or unwilling) to resolve her derived production command internally, and instead resorted to external modification, to get herself back on track. We have therefore seen how external modification can be treated as a strategy for other-monitoring; below we consider its broader implications for joint monitoring in dialogue.

## Joint monitoring in dialogue

This last example occurred in the context of a dialogue in which an addressee queried her interlocutor. However, there is also a sense in which successful dialogue depends on a somewhat different kind of monitoring than monologue. In this section, we consider the monitoring of sequential contributions to dialogue and then turn to concurrent feedback. Finally, we discuss the way in which alignment facilitates monitoring and then consider the monitoring of alignment itself.

### Monitoring of sequential contributions to dialogue

When *A* reaches *to* in the utterance *I would like to drink a beer* (time *t*), *A* predicts that *A* will utter *drink* at *t* + 1 and *a beer* at *t* + 2, and *B* also predicts *A*’s utterance at *t* + 1 and *t* + 2 (though in general *B’*s predictions will be less accurate than *A*’s). These predictions form the basis for self- and other-monitoring as discussed above.

Now consider a simple dialogue consisting of a question and an answer:

(2) A: What would you like to drink?

B: A beer, please.

We can think of this pair of contributions as distributing a single utterance over two interlocutors. After *A* says *to* at time *t*, *A* predicts that she will utter *drink* at *t* + 1, and also that *B* will respond at *t* + 2. Similarly, *B* predicts what *A* will say at *t* + 1 and will use this to predict his response at *t* + 2. Thus, both *A* and *B*’s predictions are also distributed across both participants.

As Pickering and Garrod (2013) pointed out, a well-coordinated dialogue such as a question followed by an appropriate answer is as coherent as an utterance by a single speaker. Thus making predictions about such a dialogue is of similar complexity to making predictions about an utterance by a single speaker. Hence forming such predictions in dialogue should not, in principle, be harder than forming predictions in monologue because they are sequential—the only complication is that the predictions need to be “tagged” with the person that they are predictions about. These predictions therefore allow both participants to monitor dialogue, just as they can monitor monologue. When *A* is speaking, *A* uses self-monitoring (Figure 1) and *B* uses other-monitoring (Figure 2); when *B* is speaking, *A* uses other-monitoring and *B* uses self-monitoring.

Although the mechanisms for this type of dialogue are similar to those in monologue, the ways in which they are used is different. When *A* utters *What would you like to drink?*, *A* realizes that *B*’s response is highly constrained by *A*’s question, the situational context, and the assumption that *B* will obey the conventions of adjacency pairs (Sacks et al., 1974)—primarily, that a direct question requires a timely, relevant answer (or something that queries the question). If *A* and *B* are in a pub, then *A* predicts that *B* will respond with an alcoholic or soft drink (from a limited set). So questioners make strong predictions about the timing and linguistic properties of the response (e.g., 0–2 s, DRINK, noun phrase). Moreover, the questioner “plants” a type of production command in the answerer and uses her derivation of that command to drive her forward modeling of answerer’s predicted response. It is therefore clear how the questioner can tightly integrate her own and the answerer’s production commands. The answerer also predicts that his response is constrained by the question. In our example, *B* derives *A*’s production command and the associated predicted response (alcoholic or soft drink). Assuming that *B* follows convention, he is constrained to produce a response that is compatible with this prediction. Like the questioner, the answerer tightly integrates both production commands.

To explicate the process of monitoring, let us consider different situations. At a dinner party, *A* might predict that *B* would respond with *red wine* or *white wine—*this is *A*’s derived representation of *B*’s production command. In our example, *B*’s response might therefore lead to a minor discrepancy. At this point, *A* would compute the discrepancy (informally, wine minus beer) and use this to drive the inverse model to modify the derived production command. But at a tea party, *B’*s response might be sufficiently discrepant from *A*’s prediction (*white tea or black tea*) that *A* would not be able to use the inverse model to modify the derived production command and would instead leave the language processing mechanism. Such a discrepant response would presumably activate mechanisms associated with theory of mind and general-purpose conflict resolution (see Internal Modification section). In conclusion, the mechanisms of self- and other-monitoring apply to such forms of dialogue, and become interwoven just as speaking and listening become interwoven.

We propose a similar account for other types of split utterance. For example, a speaker can encounter difficulties producing a complete utterance, perhaps because of problems with lexical selection. In such cases, an addressee can then provide a missing component by proxy (*A: That tree has … uh …uh*; *B: tentworms*; Clark and Wilkes-Gibbs, 1986). It may be that *A* is soliciting a particular type of response, and if *B* realizes this, then the situation is closely analogous to question-and-answer. Alternatively, *A* may not be soliciting a response, in which case *B’*s interjection is more surprising. If so, the discrepancy will tend to be large and *A* will be more likely to face a conflict. Such conflict is also likely in cases of true interruption, or when the addressee produces a hostile continuation to make a point (e.g., *A: In fact what this shows is. B: that you are an idiot*; Gregoromichelaki et al., 2013).

### Concurrent feedback and monitoring

As noted in Section External Modification, speakers can be affected by addressees’ concurrent verbal and non-verbal feedback, including looks of puzzlement or surprise, queries, and acknowledgments. We propose that the speaker does not treat such feedback as a separate message involving an independent derived production command but rather integrates the feedback into her own production command via monitoring. This form of monitoring involves input from the addressee (and is in that sense a form of other-monitoring) but affects the speaker’s own production command. In other words, the speaker does not (primarily at least) monitor the feedback itself but rather treats the feedback as an external input to self-monitoring and repair.

Let us return to Figure 1. The speaker performs self-monitoring by comparing the predicted utterance percept with the utterance percept to produce a discrepancy that is then input into an updated production command. We assume that the addressee’s feedback affects the mechanisms involved in self-monitoring. A confirmation (*Yeah, OK*, head nodding, etc.) constitutes positive feedback which does not affect the discrepancy or may even reduce it (e.g., a signal of understanding when an utterance is produced in noise). But a query causes the speaker to revisit the process of monitoring and, most likely, leads to a higher discrepancy, which in turn makes it more likely that the speaker will reformulate her production command. Note that the persistent absence of positive feedback may appear sufficiently unusual to serve as an indication of some form of difficulty (Bavelas et al., 2000).

Following a query, the speaker needs to identify the location and cause of difficulty. Speakers are presumably aware of the likely lag between an utterance and an addressee’s indication of misunderstanding (if it occurs) and they can therefore determine the locus of difficulty. They can also use the type of feedback as an indication of the cause of difficulty. Consider (3), from Horton and Gerrig (2005).

(3) A: and um it- you know it’s rea- it’s it was really good and of course she teaches theology that was another thing

B: mm

A: I- m- I- Isabelle

B: oh that’s great.

Here, *A* treats *B*’s *mm* as an indication of failure to understand, and presumably infers the locus as the underspecified referring expression *she*. *A* responds by switching to the more explicit *Isabelle*, a strategy which appears to be successful. In this example, the large discrepancy resulting from *B*’s *mm* generated an inverse model that led to reformulation of *A*’s production command (in this case, the command to produce explicit reference). A similar situation is illustrated in (1), where Lesley’s interjection *Forty nine wha:t?* involves some reduplication of the original utterance in a way that can help Hal’s reformulation.

### Monitoring and alignment

Pickering and Garrod (2004) pointed out that interlocutors tend to align with each other, for example choosing the same words, syntactic structures, or phonology as each other. Such alignment underlies conversational success, which occurs when interlocutors align their understanding of a situation (to a sufficient extent) and provides an explanation of why interlocutors are not overcome by the complexity of coordinating their activity with another person (Garrod and Pickering, 2004).

Alignment clearly assists other-monitoring, because it means that the comprehender’s predictions about the speaker’s upcoming utterance are more likely to be correct. Informally, if I am sufficiently similar to you, I can predict your behavior by predicting what I would do myself, and the same is as true for language as any other action. When the comprehender is different from the speaker, he can accommodate to differences between them, but of course such adaptation is likely to be imperfect (because of limited knowledge of his partner and potential egocentric biases). The more similar the interlocutors are, the less this will be necessary. For example, a comprehender may be unable to predict whether a speaker will utter *chef* or *cook*; but after one or both of them has used the term *cook*, the comprehender can now predict that the speaker will utter *cook*. (In fact, alignment may make the speaker more confident that she will utter *cook* as well.)

Of course, the speaker is actually more likely to utter *cook* under these circumstances. Thus the comprehender experiences a reduction in uncertainty in both the utterance percept and the predicted utterance percept. This also leads to a reduction in discrepancy, and so the comprehender is more likely to construct an accurate derived production command and not have to leave the language processing system (or engage in extensive conflict-monitoring). In turn, co-composition and feedback are more likely to be supportive and confirmatory—interlocutors will tend to contribute to a successful joint activity.

One way in which monitoring can help alignment has just been noted: Interjections that result from other-monitoring often involve some repetition. Thus, *Forty nine wha:t?* repeats two words and the syntactic structure of *forty nine g’rillas*. It may be that the Lesley is aligning with Hal’s utterance (i.e., via priming), or it may be that the form of her interjection is designed to facilitate Hal’s repair (so that Hal is more likely to realize the nature of the problem). According to Pickering and Garrod (2004), linguistic repetition enhances alignment.

Finally, interlocutors can use the output of other-monitoring to estimate the success or otherwise of an interaction. (It is possible that the output of self-monitoring can also be used to some extent.) Informally, if the comprehender makes accurate predictions about the speaker’s utterances, he will assume that the conversation is “flowing” well. In other words, if the discrepancies are consistently low, the interaction appears to be successful, and he will conclude that he is likely to be well-aligned with the speaker. As successful conversations involve an increase in alignment, he will in fact expect the discrepancy to reduce over time. If this does not happen, the comprehender will tend to focus on repair strategies (e.g., more clear indications of misunderstanding) or make an explicit attempt to “restart” (e.g., “Let’s try another tack”), something which might prove particularly frequent in arguments and adversarial negotiation. In general, we can regard this potentially long-term use of other-monitoring (e.g., to determine change of discrepancy over time) as involving metacognitive processes that may in themselves emphasize mentalizing.

## Conclusion

In this paper, we have provided an account of monitoring that applies to both language production (self-monitoring) and language comprehension (other-monitoring), and to both monologue (where speaker and addressee roles are clearly distinct) and dialogue (where interlocutors play both roles). The account assumes that both comprehenders and producers predict upcoming utterances, and the process of monitoring involves comparing predicted with actual utterances. It suggests that the processes involved in predicting and monitoring self and other will engage largely the same neural systems in similar ways, and will lead to similar patterns of breakdown. Our paper seeks to explicate the process of monitoring, and assumes that people compute the discrepancy between the predicted and actual utterances and use this to modify the command that underlies the producer’s utterance (the production command) or the comprehender’s assumption about the producer’s utterance (the derived production command). Such modification is sometimes straightforward, but sometimes requires the comprehender (or, more rarely, the producer) to construct a new command. We propose that dialogue tends to reduce the difficulty of such modification, because interlocutors tend to become aligned to each other. In conclusion, our account seeks to provide a unified explanation of monitoring, but we argue that it also shows how monitoring is not a peripheral component of language processing but rather central to everyday language use.

## Conflict of interest statement

The authors declare that the research was conducted in the absence of any commercial or financial relationships that could be construed as a potential conflict of interest.

## Footnotes

^1^In fact, the inverse model is already used to derive the production command which leads to original prediction (see Figure 6 of Pickering and Garrod, 2013). This “original” use of the inverse model is based on the utterance percept alone as there is no predicted utterance percept (and hence the utterance percept equals the discrepancy). Our new use of the inverse model in fact leads to the derived production command *i_B_*(*t* + 1) which in turn leads to the derived production command *i_B_*(*t* + 1), which is then used to update the prediction. (Note also that we assume that the context remains constant.)

^2^We assume that speakers are very confident about their own implemented representations during self-monitoring. They may have “privileged access” to their utterance percept, or the chances of internal misperception of their own implemented representations is negligible. In contrast, they may misperceive their own articulated utterance, when using comprehension-based “external loop” monitoring. Under most conditions, we expect the discrepancy to be larger in comprehension than production (but see Joint Monitoring in Dialogue section for qualification).

^3^(.) indicates brief pause; [indicates overlap; : indicates lengthening; .*hh* indicates long inhalation. We have added bold font for illustrative purposes.
